# Structural Insight into the Contributions of the N-Terminus and Key Active-Site Residues to the Catalytic Efficiency of Glutamine Synthetase 2

**DOI:** 10.3390/biom10121671

**Published:** 2020-12-14

**Authors:** Wen-Ting Chen, Hsin-Yu Yang, Chih-Yu Lin, Yi-Zong Lee, Szu-Chin Ma, Wei-Cheng Chen, Hsien-Sheng Yin

**Affiliations:** Institute of Bioinformatics and Structural Biology, and College of Life Sciences, National Tsing Hua University, No. 101, Section 2, Kuang-Fu Road, Hsinchu 30013, Taiwan; thg39421@yahoo.com.tw (W.-T.C.); yanghanfish@gmail.com (H.-Y.Y.); jenny211207@gmail.com (C.-Y.L.); s942645@go.thu.edu.tw (Y.-Z.L.); nightmare28.loveu@gmail.com (S.-C.M.); criedad@gmail.com (W.-C.C.)

**Keywords:** glutamine synthetase, *Drosophila melanogaster*, enzyme kinetics, crystal structure, circular dichroism

## Abstract

Glutamine synthetase (GS) catalyzes the condensation of ammonia and glutamate, along with ATP, to form glutamine. Despite extensive studies on GSs from eukaryotes and prokaryotes, the roles of the N-terminus and other structural features in catalysis remain unclear. Here we report the decameric structure of *Drosophila melanogaster* GS 2 (DmGS2). The N-terminal short helices, α1 and α2, constitute a meander region, and form hydrogen bonds with residues 3–5 in the N-terminal loop, which are not present in the GSs of other species. Deletion of α1 or α1-α2 inactivates DmGS2. Notably, the Arg4 in each monomer of one pentamer forms hydrogen bonds with Glu7, and Asp8 in the adjacent monomer of the other pentamer. Replacement of Arg4 with Asp (R4D) abolishes activity. Analytical ultracentrifugation revealed that Arg4 is crucial for oligomerization. Circular dichroism spectra revealed that R4D may alter the secondary structure. We mutated key residues to identify the substrate-binding site. As Glu140 binds glutamate and Glu311 binds ammonia, mutants E140A and E311A have little activity. Conversely, mutant P214A (P contributes to ATP binding) has higher activity than wild-type DmGS2. These findings expand the understanding of the structural and functional features of the N-terminal meander region of DmGS2 and the residues important for catalytic efficiency.

## 1. Introduction

Glutamine synthetase (GS) plays a crucial role in nitrogen metabolism by catalyzing the ATP-dependent condensation of glutamate with ammonia to produce glutamine, ADP, and inorganic phosphate [1]. In the human brain, GS modulates the cellular level of harmful ammonia, and converts neurotoxic glutamate into nontoxic glutamine [2]. In addition, Alzheimer’s disease patients have elevated GS in their cerebral spinal fluid [3]. In plants and bacteria, the glutamine produced by GS is used as a nitrogen source for metabolism [4,5]. *Drosophila melanogaster* GS (DmGS) has two forms, namely, isoform I (DmGS1) and isoform II (DmGS2) [6,7]. The mitochondrial DmGS1 predominates in larva, and its mRNA is highly expressed in the epidermis. The cytoplasmic DmGS2 is the most abundant form in adult flies, comprising 90% of total GS activity [8,9]. In the relatively late embryonic stage, DmGS2 mRNA is expressed in the nervous system and accumulates in axon bundles, correlating with synapse formation [7]. Recent studies have indicated that GS2 is expressed in the glial cells of the young Drosophila mushroom body [10]. Glial cells take up glutamate released from the synapses and converts it to glutamine via GS. The mushroom body of the Drosophila brain also plays vital roles in olfactory memory and learning [11]. Consequently, Drosophila GS2 may be critical for learning and memory.

Several studies have characterized the structure and function of eukaryotic and prokaryotic GSs [2,12,13,14]. The three-dimensional structure of eukaryotic GS has revealed a decamer consisting of two stacked pentamers. Thus, eukaryotic GSs have 10 active sites, each of which is located at a monomer–monomer interface. In human and dog GSs, the N-terminal β-grasp domain of one monomer connects with the β-sheet of the C-terminal catalytic domain in the next monomer, constituting a funnel-shaped pocket [2]. Bacterial GS structures are dodecamers consisting of two stacked hexamers, with 12 active sites formed between subunits [15]. Each active site also contains a funnel-shaped pocket, having an entrance for the substrates ATP and glutamate [1]. Despite extensive studies on GSs from eukaryotes and prokaryotes, the roles of the N-terminus and other structural features in catalysis remain unclear. Herein, we report the X-ray crystallographic structure of DmGS2 in the ADP-bound state. Our results provide the first structural evidence for GSs, with respect to the specific interaction of each N-terminal meander region with its nearest-neighbor monomer. Overall, the results fill vital gaps in our understanding of the structural impact and roles of the N-terminal meander region and catalytic residues on the structure of DmGS2.

## 2. Materials and Methods

### 2.1. Materials

Ampicillin, imidazole, NaCl, and tris(hydroxymethyl)aminomethane (Tris) were supplied by USB (Cleveland, OH, USA). ADP, glutamine, ATP, potassium arsenate, MgCl_2_, FeCl_3_, HCl, (NH_4_)_2_SO_4_, 2, 4, 6-trichloroanisole, fomblin oil, and 2-mercaptoethanesulphonic acid were purchased from Sigma-Aldrich (St. Louis, MO, USA). Li_2_SO_4_ monohydrate, ADA, polyethylene glycol 4000, and 2-propanol were obtained from Hampton research (Aliso Viejo, CA, USA). *Escherichia coli* BL21 (DE3) strain was obtained from Yeastern Biotech (Taiwan). Isopropyl β-D-1-thiogalactopyranoside was purchased from Protech (Taiwan).

#### 2.1.1. Expression and Purification of DmGS2

The full-length cDNA of mature DmGS2 (GenBank accession number X52759) was cloned into vector pUC57 (Genomics BioSci & Tech, Taipei, Taiwan). The codons for residues E140, P214, and E311 were each replaced with that for an alanine, and R4 was mutated to aspartate using Quikchange site-directed mutagenesis kit reagents (Stratagene, Amsterdam, The Netherlands). The N-terminal 13 residues were deleted (Figure 2). Wild-type (WT) and mutated DmGS2 genes were individually cloned into pET-28a(+) (Novagen, Whitehouse Station, NJ, USA), with an upstream T7 promoter-His6 tag, and expressed in E. coli BL21 (DE3). The gene sequences were confirmed by DNA sequencing (Mission Biotechnology Inc., Taiwan). Supplementary Table S1 lists the primers used.

#### 2.1.2. Bacteria

Cells were cultured in Luria–Bertani medium, containing 50 μg/mL ampicillin. When the OD_600_ reached 0.6, isopropyl-thio-β-D-galactopyranoside (0.4 mM final concentration) was added into each culture to induce protein expression. After growth for 16 h at 20 °C, whole cells were harvested by centrifugation, and lysed by sonication in 25 mM Tris-HCl, 100 mM NaCl, pH 7.4, then centrifuged at 100,000× *g* for 30 min at 4 °C. The His-tagged proteins were purified by Co^2+^-affinity column chromatography (BD Biosciences, CA). The column was first washed with 100 mM imidazole, 25 mM Tris-HCl, 100 mM NaCl, pH 7.4, and protein was eluted in the same solution but containing 300 mM imidazole. A Centricon YM-10 centrifugal filter device (Millipore, MA, USA) was then used to remove imidazole, and to concentrate each protein. The purity of the purified protein products was assessed by SDS-PAGE [16], and peptide mass fingerprints were obtained using an Autoflex III MALDI-TOF mass spectrometer (Bruker Daltonics Inc., Billerica, MA, USA) [17]. Protein concentrations were determined using Quick Start Bradford Protein Assay reagents (Bio-Rad, Hercules, CA, USA), with bovine serum albumin as the standard. DmGS2 (15 mg/mL) in 25 mM Tris-HCl, 100 mM NaCl, pH 7.4 served as stock solutions.

### 2.2. Crystallisation, Data Collection, and Refinement

Crystals of WT DmGS2 were grown in greased wells of 48-well plates (Hampton Research, Aliso Viejo, CA, USA) using the hanging-drop vapor diffusion crystallization method and protein (12 mg/mL) in 20 mM Tris-HCl, pH 7.9, 150 mM NaCl, 5 mM MgCl_2_, 5 mM ATP, and 1 mM sodium 2-mercaptoethanesulphonate. A single crystal was grown in 0.1 M Li_2_SO_4_ monohydrate, 0.1 M ADA pH 6.5, containing 12% *w/v* polyethylene glycol 4000 and 2-propanol (2%) and was then soaked in cryoprotectant (fomblin oil), and subsequently frozen in liquid nitrogen for data collection. X-ray diffraction data were collected at the SPXF beamline BL13B1 of the National Synchrotron Radiation Research Center in Hsinchu, Taiwan, using a mar345 Image Plate Detector (Marresearch GmbH, Germany). Structure calculation, refinement, and validation were as previously described [18,19]. Refinement statistics are summarized in Table 1. Diffraction data were collected for WT DmGS2 to 2.12 Å resolution. Diffraction datasets were processed using the HKL-2000 package (HKL Research Inc., Charlottesville, VA, USA) [20]. Table 1 summarizes unit-cell dimensions, data collection, and crystallographic parameters.

Molecular replacement was performed using Molrep in the CCP4 software suite [21] and, as the search model, the complete human GS structure (Protein Data Bank (PDB) ID 2OJW) [2], which shares 65% sequence identity with DmGS2. The initial model was refined using Refmac5 [22], and rebuilt with Coot [23]. Structure validation was performed by PROCHECK v.3.5.4 [24], and secondary structures were identified using DSSP [25]. The atomic coordinates and structure factors for DmGS2 were deposited in the PDB under accession code 7CPR. The molecular graphics software PyMOL (DeLano Scientific; http://www.pymol.org) and Discovery Studio 3.5 (Accelrys Inc., San Diego, CA, USA) were used for molecular visualization.

### 2.3. Enzyme Assays

The transferase activity of WT DmGS2 and mutants (5 μM) was measured as described [26], and the biosynthetic activity of each was also measured [27]. Each transferase reaction contained 40 mM imidazole (pH 7.9), 90 mM glutamine, 30 mM hydroxylamine, 3 mM MnCl_2_, 0.4 mM ADP, and 20 mM potassium arsenate, with incubation at 37 °C in 0.5 mL. After 15 min, the reaction was stopped by adding 1 mL of a mixture of 3.3% FeCl_3_, 2% trichloroacetic acid, and 0.25 N HCl, and absorbance at 535 nm was recorded (U-3300 UV-VIS spectrophotometer, Hitachi, Tokyo, Japan) against a blank identical to the above, except lacking ADP. The amount of γ-glutamyl hydroxamate was determined by the increase in absorbance. All measurements were within the linear range of the γ-glutamyl hydroxamate standard curve. Each biosynthetic reaction contained 50 mM imidazole-HCl (pH 7.0), 12 mM ATP, 50 mM MgCl_2_, 100 mM L-glutamate, and 50 mM NH_4_Cl in a final volume of 100 μL. The reaction was initiated by adding enzyme solution, with subsequent incubation at 37 °C for 30 min. The reaction was stopped by adding 285 μL of L-ascorbic acid (12% *w/v* in 1 N HCl) and 15 μL of 0.1 M (NH_4_)6Mo_7_O_24_·4H_2_O (in 0.3 N H_2_SO_4_) to generate color for 5 min. Finally, 300 μL of 2% (*w*/*v*) sodium citrate in 2% (*v*/*v*) acetic acid was added to quench further color development, and the absorbance was measured at 655 nm.

### 2.4. Kinetic Analysis

UV-visible data were converted to initial velocity using Excel 2010 (Microsoft), and kinetic parameters were determined by fitting the data to the Michaelis–Menten equation using nonlinear regression and Prism software version 6.01 for Windows (GraphPad Software, La Jolla, CA, USA).

### 2.5. Analytical Ultracentrifugation

To determine the oligomerization state of WT DmGS2 and its mutant R4D, samples (500 μL, 1.0 mg/mL) were subjected to sedimentation velocity at 20 °C using a Beckman XL-A Optima analytical ultracentrifuge, equipped with an absorbance optics unit (280 nm) and a Ti-60a titanium rotor. The program, SEDFIT85, was used to calculate the sedimentation coefficient [28].

### 2.6. Biophysical Properties of WT DmGS2 and R4D

Circular dichroism (CD) spectra were analyzed to estimate protein secondary structure and stability using an Aviv 202 spectropolarimeter (Aviv Biomedical Inc., Lakewood, NJ, USA) [29]. WT DmGS2 or mutant R4D proteins (15 µM) in 10 mM potassium phosphate buffer (pH 7.4) were measured at 25 °C in the far-UV region (195–260 nm) using a 1-mm path-length cuvette. Three CD scans were averaged, and spectra are reported as mean residue ellipticity (ɵ), in deg cm^2^ dmol^−1^.

## 3. Results and Discussion

### 3.1. Overall structure of DmGS2

**Figure 1:**
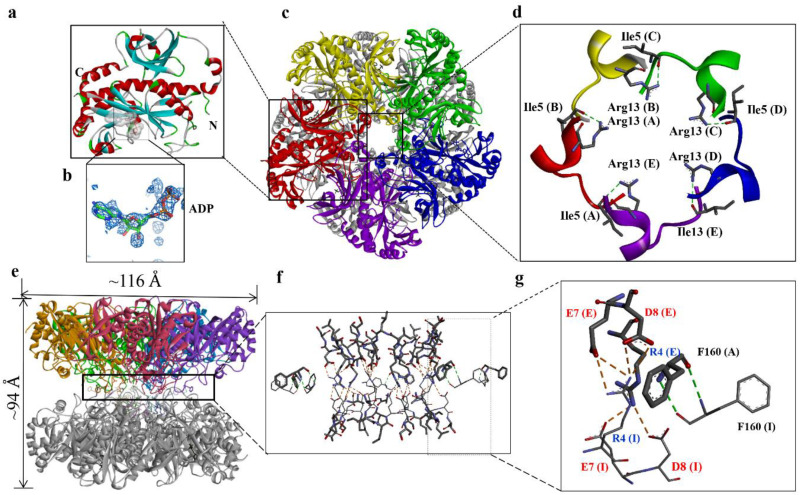
Three-dimensional structure of DmGS2. Ribbon diagrams for the DmGS2 monomer (**a**) and decamer (**c**). (**b**) The |Fo-Fc| electron density map contoured at 3 σ is drawn for the ADP molecule. One pentamer is shown in grey, and the monomers of the other pentamer are shown with different colors: red, chain A; yellow, chain B; green, chain C; blue, chain D; purple, chain E. (**d**) A typical interaction at the subunit–subunit interface of the N-termini. Residue 5 from one subunit forms an H-bond with residue 13 in the nearest-neighbor subunit, e.g., residue 5 of chain A H-bonds with residue 13 of chain E, etc. (**e**) Side view of the pentamer–pentamer contacts shown in (**c**). (**f**,**g**) Close-up view of H-bonding interactions between the pentamers. Red denotes negatively charged residues, and blue denotes positively charged residues. Hydrogen bonds are formed between Arg4 in each subunit of one pentamer and Glu7 and Arg8 in the nearest-neighbor subunit of the other pentamer. Interpentamer H-bonds between the main-chain atoms of Phe160 residues in opposing protomers are also seen at the pentamer–pentamer interface.

A structure was obtained for the DmGS2 decamer in complex with ADP (Figure 1). The final decameric model comprised two face-to-face pentameric rings with 2-fold, non-crystallographic symmetry (Figure 1b,d). Each DmGS2 monomer contained 367 residues, and ADP, although two residues that were engineered for a T7 tag and the His6 sequence were missing. The stereochemical quality of the crystal structure (Table 1) was examined with SFCHECK [30], revealing that the ϕ-ψ angles for 99.9% of residues were in regions allowed by the Ramachandran plot [31]. Each monomer contained a so-called β-grasp domain in the N-terminal portion and eight-stranded β-sheets as the catalytic domain in the C-terminal region (Figure 1a), similar to structures of other eukaryotic GSs [2,12,13]. ATP and Mg^2+^ were included in the crystallization mixture; the |Fo-Fc| electron density contoured at 3 σ map showed for the ADP molecule rather than ATP (Figure 1b). The hydrolysis of ATP may be promoted by Mg^2+^, which accelerates the reaction in aqueous solution [32,33]. Like the crystal structures of other eukaryotic GSs, the decamer of DmGS2 in complex with ADP (Figure 1c) consists of two face-to-face pentameric rings, which are related by 2-fold non-crystallographic symmetry [2,12,13]. Notably, in one pentamer (chains A to E), I5 located in that loop formed a hydrogen bond (H-bond) with R13 in a short α-helix in the nearest-neighbor chain (Figure 1d). Overall, the DmGS2 decamer was approximately spherical, with a diameter of ~116 Å along the fivefold axis, and ~94 Å along the twofold axis (Figure 1c,e). Each subunit interacted with two neighboring subunits along the fivefold axis. Moreover, an interpentamer H-bond between the main-chain atoms of Phe160 residues in opposing protomers was found at the pentamer interface (Figure 1f). All residue and H-bonding interactions were conserved in other GSs [2]. Notably, the interactions between DmGS2 pentamers were more numerous compared with other GSs. Arg4 in each subunit of one pentamer also formed H-bonds with Glu7 and Asp8 in each subunit of the other pentamer (Figure 1f). These specific residues were not present in other eukaryotic and prokaryotic GSs [2,12,34,35]. The additional charge–charge interactions between the DmGS2 pentamers (i.e., those not observed in other GSs) may play an important role in decamer formation.

### 3.2. Quaternary Structure of DmGS2

**Figure 2:**
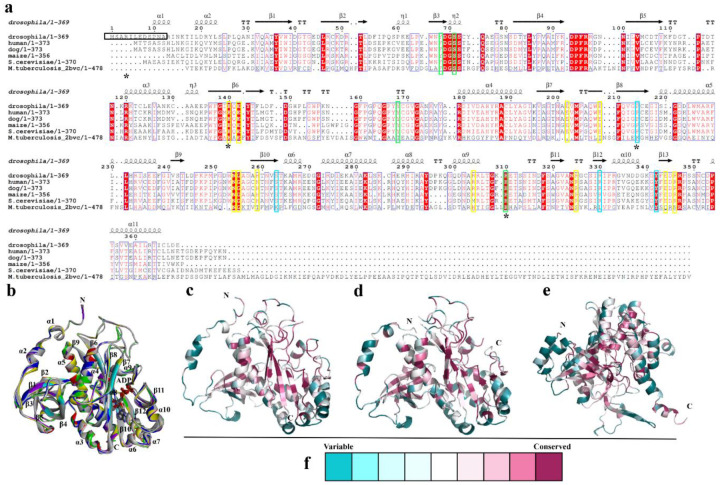
Amino-acid sequence alignment and structural comparison of GSs. (**a**) Structure-based sequence alignment of GSs from D. melanogaster (PDB #7CPR), human (#2QC8), dog (#2UU7), maize (#2D3A), S. cerevisiae (#3FKY), and M. tuberculosis (#2BVC), created using the crystal structure of DmGS2 as a template. The secondary structure elements and residue numbering shown above the sequences refer to D. melanogaster GS: α, α-helix; η, η-helix; β, β-sheet; TT, β-turn. Conserved residues are indicated as white letters on a red background, partially conserved residues are indicated as red letters, and similar residues are indicated as blue boxes. Residues involved in binding of ammonia (green box), ADP (turquoise box), and glutamate (yellow box) are highlighted. Asterisks denote DmGS2 residues R4, E140, P214, and E311 mutated for this study. The horizontal black box denotes DmGS2 residues 1 to 13 truncated for this study. The sequence alignment was generated with the Clustal Omega [41], and the figure generated with ESPript [42]. (**b**) Overlay of 10 monomeric structures of DmGS2. Secondary structure elements included (α, helix; β, sheet) and are labeled according to their position in the structure (α1 to α10; β1 to β11). ConSurf analysis of the glutamine synthetase from D. melanogaster (PDB #7CPR) (**c**), human (#2QC8) (**d**) and M. tuberculosis (#2BVC). (**c**,**e**) The structure of N-terminal meander regions (such as residues 3–29 in D. melanogaster and residues 3–24 in human) are highly variable. The inner core of N-terminal β-grasp domain (such as residues 30–117 in D. melanogaster and residues 25–112 in human) and C-terminal catalytic domain (such as residues 118–369 and residues 113–373 in human) are highly conserved. (**f**) Molecule colored by the new color-blind friendly scale.

**Figure 3:**
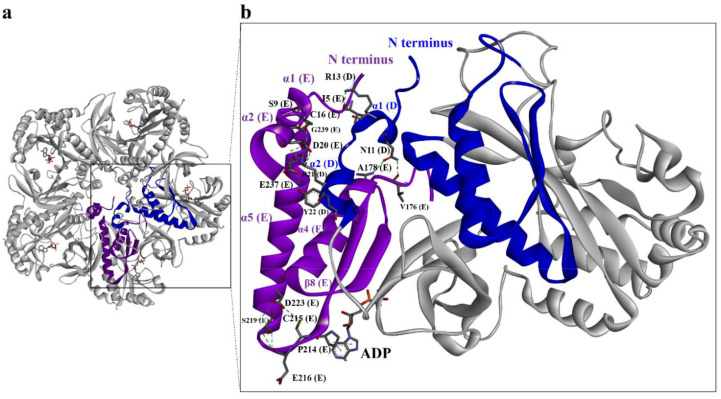
N-terminal meander region interactions at the subunit–subunit interface within one DmGS2 pentamer. Two short α-helices (α1, α2) at the N-terminus of GS constitute the “meander region” of DmGS2. (**a**) Ribbon diagram of the DmGS2 pentamer. (**b**) The N-terminal meander region of one subunit (e.g., chain E) is shown as a purple ribbon that forms H-bonds with the nearest-neighbor subunit (chain D), shown in blue.

Structure-based sequence alignments (Figure 2a) indicated that residues involved in the binding of substrates are conserved in the GSs of many species [2,36,37]. In humans and dogs, for example, D63, S66, Y162, and E305 bind ammonia, and P208, S257, R324, and Y336 are involved in ADP binding. In addition, 10 other residues are involved in glutamate binding [1,2] (Figure 2a). Thus, these residues likely play important roles in catalysis, and they are also presented in DmGS2 (Figure 2a). Within the decameric structure of DmGS2, each monomer bound ADP with identical protein-ADP interactions (Figure 2b). Secondary structural elements included α-helices and β-sheets that are labelled according to their position in the amino acid sequence (α1 to α10; β1 to β11) (Figure 2a,b). Close examination revealed differences between DmGS2 and human GS in the N-terminal region. DmGS2 had six additional N-terminal residues (NSARIL) (Figure 2a–c) that formed a turn not being found in other GSs (Figure 2d,e) [2,15,38]. The short helices, α-1 and α-2, the so-called meander region, located in the N-terminal region of DmGS2 (Figure 2b and Figure 3b) was highly variable and also observed in other GSs, such as human and M. tuberculosis using ConSurf analysis [39] (Figure 2d,e). In addition, the inner regions of the N-terminal β-grasp domain (such as residues 30-117 in D. melanogaster and residues 25–112 in human) and the C-terminal catalytic domain (such as residues 118–369 and residues 113–373 in human) are highly conserved (Figure 2c,d). In addition, the meander region also presented in maize (Figure 2a) (residues 1–17) GSs [12]. Compared with maize GS, mammalian GS (residues 3–24) had a slightly longer N-terminus (Figure 2a). The subunit–subunit interface areas of maize and mammalian GSs were measured using the EMBL-EBI PISA server [40], and were 1914.7 Å^2^ and 1886.1 Å^2^, respectively. This implied that the mammalian GS had a larger subunit–subunit contact area. This finding suggested that the meander region may play a role in stabilizing the pentameric ring [2]. Moreover, the meander region of DmGS2 made contacts with neighboring subunits in the core of the pentamer (Figure 3a). In addition, the pentameric structure of DmGS2 indicated that Asn11 and Arg13 from one subunit made H-bonds with nearest-neighbor subunits at Val176, Ala178, and Ile 5, respectively (Figure 3a,b). The meander region also existed in eukaryotic GSs, and may contribute to stabilizing the pentamer [2,32]. Thus, the data for DmGS2 are the first to reveal that the meander region can interact specifically with other regions of the GS enzyme.

### 3.3. Structural Comparison of N-Terminal Meander Region of Maize and Human GSs

**Figure 4:**
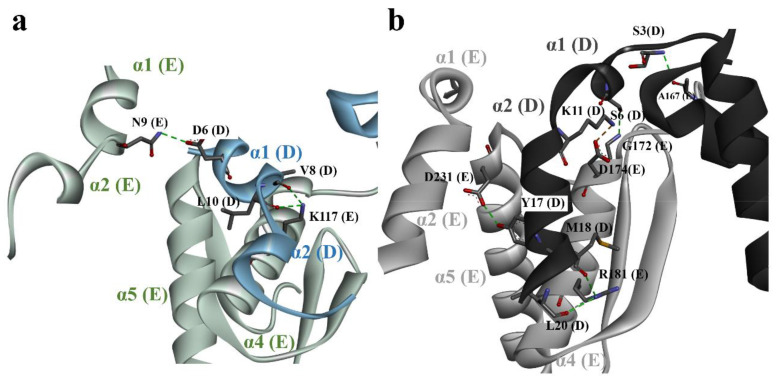
Structural comparison between the N-terminal meander region and the neighboring subunit of maize and human GSs. (**a**) N-terminal meander region of one subunit (e.g., chain D) of maize GS is shown as a blue ribbon, and forms H-bonds with the nearest-neighbor subunit (chain E), shown as a green ribbon. (**b**) Interactions involving the N-terminal meander region of one subunit (e.g., chain D, black) and nearest-neighbor subunit (e.g., chain E, gray) in human GS.

The GS family has a high percentage of amino acid sequence identity, except for the N-terminal meander region (residues 1–22) (Figure 2). There are two unique properties found in the N-terminal meander region of DmGS2, one is that residues 4–8 of 2-fold symmetry-related monomers have H-bonding interactions between Arg4 and (Glu7 and Asp8), the other is Ile5 in one monomer forms H-bonding with Arg13 in the adjacent monomer. Those interactions in the N-terminal meander region of DmGS2 may stabilize the five monomers and strengthen pentamer–pentamer interactions compared with other GSs. Although these residues in DmGS2 do not exist at corresponding positions in GSs from other species (Figure 2), plant GS residue D6 in α1 of one monomer forms H-bonds with residue N9 in α1 of the adjacent monomer (Figure 4a). The meander region (loop α1-α2) of human GS does not contact the meander regions in other monomers, but the meander region in one monomer makes close contacts with α4 and α5 of the adjacent monomer (Figure 4b). The real roles of the meander region in animal and plant GSs need further examination, such as catalytic activity analysis by site-directed mutagenesis. Notably, bacterial GSs, such as that of M. tuberculosis do not have the so-called N-terminal meander region, and the N-terminal region (1–19) does not interact with any other residues [34,43]. The core of the hexameric M. tuberculosis GS is also much larger than that of eukaryotic GSs, perhaps implying that the N-terminal region of M. tuberculosis GS may not play an important role in the stabilization of its hexameric structure. Moreover, although the meander region was found to be dispensable for determining the quaternary structure of the GS2 of Saccharomyces cerevisiae [13], evidence is lacking concerning the importance, if any, of this region for catalysis mediated by other GSs.

### 3.4. DmGS2 Mutants and Kinetic Assay

An amino-acid sequence alignment was generated for GSs of Drosophila, human, dog, plant, yeast, and bacteria (Figure 2a). Previous studies found that certain conserved residues among prokaryotic and eukaryotic GS may play vital roles in catalysis or binding of substrates and other ligands [2,34,44,45]. Arg4 in each protomer of one pentamer forms H-bonds with Glu7 and Asp8 in each monomer of the other pentamer (Figure 1f). Furthermore, Arg 13 from one subunit forms an H-bond with Ile 5 of the neighboring subunit in the other pentamer (Figure 1d). To further investigate the relative importance of the residues and regions in DmGS2 to its enzyme activity, DmGS2 mutants E140A, P214A, E311A, and R4D were prepared. Additionally, to investigate the influence of N-terminal meander on the catalytic step of glutamine formation, we hypothesized that at the N-terminal at least 13 residues are essential to stabilize the conformation in the biosynthetic process, an N-terminal truncation mutant (13 residues, Δ13) was also prepared. Individual His-tagged mutants were expressed in E. coli and purified to homogeneity by cobalt ion affinity chromatography. Each of these mutated residues is involved in substrate binding, i.e., E140 (E134 in human GS) for glutamate binding, P214 (P208 in human GS) for ATP binding, and E311 (E305 in human GS) for ammonia binding (Figure 2a). Mutants R4D and Δ13 were used to characterize the importance of the charge–charge and H-bonding interactions between the meander region and other regions of DmGS2.

**Figure 5:**
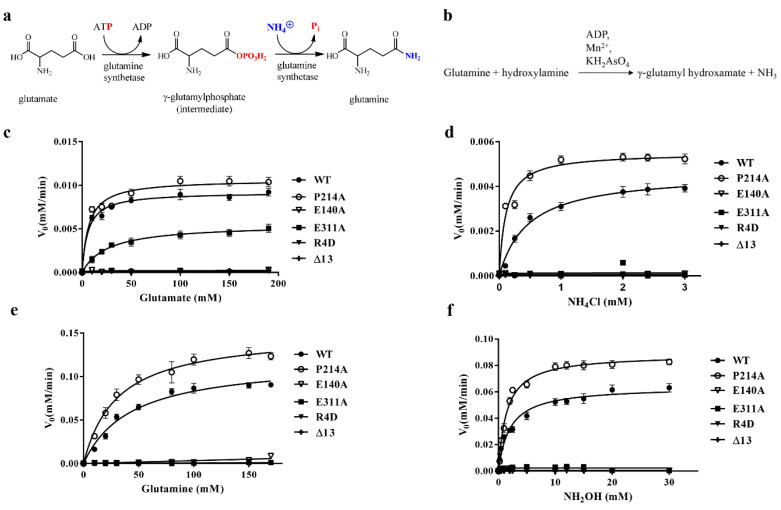
Analysis of comparative steady-state kinetics of the biosynthetic and transferase activities of wild-type (WT) DmGS2 and its mutant. (**a**) Schematic diagram of the two-step reaction catalyzed by glutamine synthetase to form glutamine via glutamate, ATP, and ammonia. The phosphate derived from ATP is colored in red, ammonium is colored in blue [46]. (**b**) The method for the estimation of GS transferase activity depends on its γ-glutamyl transferase reaction by measuring γ-glutamylhydroxamate synthesized from glutamine and hydroxylamine. (**c**,**d**) Catalytic rate for the DmGS2 biosynthetic reaction measured for various concentrations of glutamate and saturating ammonium chloride (**c**), or various concentrations of ammonium chloride and saturating glutamate (**d**). (**e**,**f**) Catalytic rate for the DmGS2 transferase reaction measured for various concentrations of glutamine and saturating hydroxylamine (e), or various concentrations of hydroxylamine and saturating glutamine (**f**). Assays were performed as described in Methods.

To assess the possible contribution of the N-terminal region to DmGS2 structure, the kinetic properties of wild-type (WT) and mutant DmGS2 were analyzed with respect to both their biosynthetic, and transferase, activities (Figure 5, Table 2 and Table 3). These results are summarized in Figure 5 and Table 2 and Table 3. The biosynthetic activity of GS is to catalyze the reaction of glutamate and ammonia to form glutamine (Figure 5a), and transferase activity is the conversion of α-glutamine to γ-glutamylhydroxamate (Figure 5b). The biosynthetic and transferase activities of the five mutants were then assessed. Neither of the two activities could be detected for mutants R4D, E140A, and Δ13 (Figure 5). In E311A case, the analysis of Michaelis–Menten kinetics did not report detectable response for using the substrates of glutamine, hydroxylamine, and ammonium chloride, indicating the loss of enzyme activity (Figure 5d–f and Table 2 and Table 3). However, E311A showed a decrease, but still substantial activity for hydrolyzing glutamate (Figure 5c). The apparent k_cat_/K_m_ values of WT and E311A for substrate glutamate were 0.34 ± 0.08 and 0.042 ± 0.005 min^−1^ mM^−1^, respectively (Table 2), indicating E311A had less catalytic efficiency than WT DmGS2. Mutant E140A had no detectable activity, indicating that E140 is important for DmGS2 function. In addition, the K_m_ and k_cat_ values for P214A were close to those of WT, with respect to the substrate glutamate; notably, for substrates glutamine, hydroxylamine, and ammonium chloride, the K_m_ values were lower than those of WT, although the corresponding k_cat_ values were higher than those of WT (Table 2 and Table 3). The results of the enzyme kinetics analysis indicated that the binding affinity and catalytic efficiency of mutant P214A were superior to WT. Moreover, residue P214 is conserved in all eukaryotic GSs [2,12] but not bacterial GS, such as the GS of M. tuberculosis [34]. Thus, this proline residue may constitute a target for screening of molecules to inhibit GS activity. Structural observations indicated that the truncation of α1-α2 in one subunit disrupted the interaction of nearest-neighbor α5, which interacted directly with the catalytic residue residing in β8 (Figure 3b). Truncation of mutant Δ13 may have destabilized the α5-β8 interface, further affecting the P214–ADP interaction, and leading to loss of the enzyme activity. Consequently, neither of the two activities could be detected for mutants R4D, E140A, and Δ13 (Figure 5).

Interestingly, DmGS2 mutant E311A had less effect on glutamate binding, but it indeed lost ammonium chloride binding ability in the biosynthetic reaction (Figure 5c,d and Table 2). In the transferase reaction, E311A lacked the transferase activity (Figure 5e,f and Table 3). This suggests that glutamine formation proceeds through a two-step mechanism: the first step entails formation of the GGP intermediate, and the second, rate-limiting step involves a nucleophilic attack by ammonia. Residue E311 is part of the ammonium-binding site that is important during the second enzymatic step [2,34]. In the biosynthetic assay, E311A could not carry out the ammonium-mediated nucleophilic attack but retained glutamate-binding ability in a saturated NH_4_Cl solution, and release of inorganic phosphate from the intermediate GGP was detectable. In the transferase reaction, the final product, γ-glutamyl hydroxamate, could not be detected, because E311A may only have weak binding affinity for NH_2_OH. The same interpretation can be used for E140A, as E140 contributes to glutamate binding. E140A had neither biosynthetic, nor transferase, activity owing to its inability to carry out the first step of the GS reaction.

### 3.5. Oligomerisation States of WT DmGS2 and R4D

**Figure 6:**
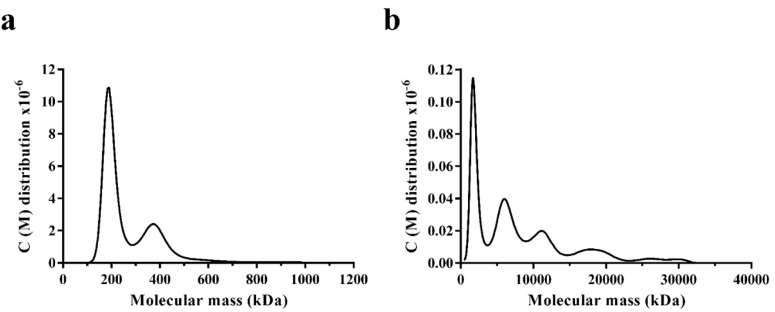
Oligomeric states of WT DmGS2 and R4D. Analytical ultracentrifugation results for WT DmGS2 (**a**) and R4D (**b**). The continuous molar mass distribution, C (M), for each protein is plotted as a function of molecular mass, and was calculated using SEDFIT85 [28].

The DmGS2 mutant R4D had no detectable GS activity. The influence of the R4D mutation on GS activity was not a consequence of the loss of a direct interaction with a substrate, because this residue is distant (30 Å) from the catalytic site. We hypothesized that the lack of activity was rather a consequence of an altered conformation of the DmGS2 decamer. To test this possibility, analytical ultracentrifugation was used to determine the oligomeric states of WT DmGS2 and R4D (Figure 6). The sedimentation coefficients for WT were 9.94 S and 14.99 S, corresponding to the respective molecular masses of 190.3 kDa (pentamer) and 377.6 kDa (decamer) (Figure 6a), as has been determined for mammalian GSs [2]. However, the sedimentation coefficients for R4D were 52.45 S, 122.48 S, 183.46 S, and 251.22 S, corresponding to respective molecular masses of 1740.8 kDa, 6100.9 kDa, 11,055.5 kDa, and 18,315.1 kDa (Figure 6b). This indicated that analytical ultracentrifugation of mutant R4D showed the unregulated large, random oligomer conformation (Figure 6b), suggesting that the huge molecular weight fractions of R4D may be due to the loss of subunit–subunit or pentamer–pentamer associations, resulting in random polymer formation. Thus, R4D was sedimented as a large, random oligomer. These data indicated that the N-terminal meander region that includes R4 is essential for pentamer formation and stability.

### 3.6. Biophysical Properties of Recombinant WT DmGS2 and R4D

**Figure 7:**
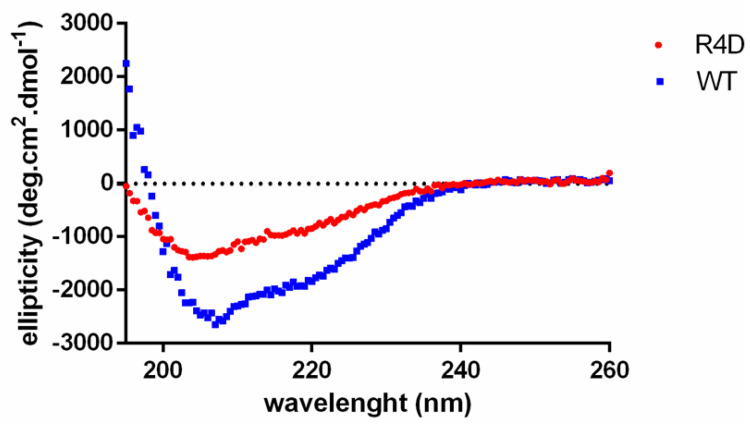
Biophysical properties of recombinant WT DmGS2 (blue) and R4D (red). Secondary structures for recombinant WT DmGS2 and R4D were measured by far-UV CD spectrum.

To clarify the secondary structure characterizations of WT DmGS2 and R4D, both were probed by CD spectroscopy. The WT DmGS2 spectra showed a minimum at ∼207 nm (Figure 7, blue line), which is consistent with the results of a previous study of pea glutamine synthetase [47]. The mutant R4D, however, exhibited spectra characteristic of a random coil structure (Figure 7, red line). These data demonstrated that the secondary structural change of R4D may account for its inability to form a stable pentameric or decameric structure (Figure 6b and Figure 7). 

## 4. Conclusions

In summary, our crystallographic data, analytical ultracentrifugation results, and enzyme activity results clearly define the specific interactions in the N-terminal meander region of DmGS2, which may contribute to both the structure, and activity, of this GS. Enzyme kinetics assays identified key residues in DmGS2 related to its enzymatic properties, and suggested a target (P214) for inhibition or potentiation of activity. Our studies represent the first evidence of the contribution of the N-terminal meander region to GS structure and function. These findings may lead to the development of new therapeutics that neutralize or enhance GS family activity.

## Figures and Tables

**Table 1:** Data collection and refinement statistics for the DmGS2/ADP complex.

	DmGS2/ADP Complex
**Data collection:**	
Wavelength (Å)	1.0000
Temperature (K)	100
Resolution (Å)	30-2.12 (2.20–2.12) ^a^
Space group	*C*121
Unit cell dimensions	
*a*, *b*, *c* (Å)	229.70, 120.92, 206.51
α, β, γ (°)	90.00, 120.09, 90.00
Number of unique reflections	234,758
Number of observations	637,222
Redundancy	2.8 (2.7) ^a^
Completeness (%)	98.0 (97.2) ^a^
Mean I/σ (I)	16.41 (2.50) ^a^
No. of protein monomers	10
Matthew coefficient (Å^3^ Da^−1^)	2.55
Solvent content (%)	51.38
Wilson *B*-factor (Å^2^)	42.2
*R*_merge_	0.071 (0.592) ^a^
**Refinement:**	
Resolution range (Å)	27.40–2.12 (2.20–2.12) ^a^
Number of reflections used	216,390 (11,245) ^a^
Number of protein heavy atoms	29,527
Number of water molecules	521
Number of heterogeneous molecules	280
Average *B*-factor (Å^2^)	50.32
*R*_work_/*R*_free_ (%)	19.19/24.08 (28.9/32.2) ^a^
**Ramachandran plot statistics:**	
Residues in favored region (%)	95.9
Residues in allowed region (%)	4.0
Residues in outlier region (%)	0.1
Average RMSD, bond length (Å)	0.016
Average RMSD, bond angle (°)	1.801
PDB entry	7CPR

^a^ Numbers in parenthesis correspond to the highest resolution shell (2.20–2.12) Å.

**Table 2:** Comparison of the biosynthetic activity of WT DmGS2 and its mutants.

Protein Type	Substrate	K_m_ (mM)	*v*_max_ (mM min^−1^) ^c^	*k*_cat_ (min^−1^)	*k*_cat_*/*K_m_ (min^−1^ mM^−1^)
WT	Glutamate ^a^	5.9 ± 1.6	0.0092 ± 0.0004	1.84 ± 0.08	0.34 ± 0.08
R4D	Glutamate	ND	ND	ND	ND
Δ13	Glutamate	ND	ND	ND	ND
E140A	Glutamate	ND	ND	ND	ND
P214A	Glutamate	6.8 ± 2.2	0.0106 ± 0.0006	2.13 ± 0.11	0.38 ± 0.13
E311A	Glutamate	26.2 ± 2.2	0.0055 ± 0.0004	1.10 ± 0.08	0.0420 ± 0.0005
WT	Ammonium chloride ^b^	0.47 ± 0.03	0.0046 ± 0.0001	0.92 ± 0.02	1.96 ± 0.08
R4D	Ammonium chloride	ND	ND	ND	ND
Δ13	Ammonium chloride	ND	ND	ND	ND
E140A	Ammonium chloride	ND	ND	ND	ND
P214A	Ammonium chloride	0.11 ± 0.03	0.0055 ± 0.0003	1.10 ± 0.06	10.6 ± 2.4
E311A	Ammonium chloride	ND	ND	ND	ND

ND, not detectable, ^a^ Parameters determined at a saturating concentration of ammonium chloride, ^b^ Parameters determined at a saturating concentration of glutamate, ^c^ The amount of enzyme used was 5 µM.

**Table 3:** Comparison of the transferase activity of WT DmGS2 and its mutants.

Protein Type	Substrate	K_m_ (mM)	*v*_max_ (mM min^−1^) ^c^	*k*_cat_ (min^−1^)	*k*_cat_/K_m_ (min^−1^ mM^−1^)
WT	Glutamine ^a^	43.1 ± 9.7	0.12 ± 0.01	24 ± 2	0.58 ± 0.08
R4D	Glutamine	ND	ND	ND	ND
Δ13	Glutamine	ND	ND	ND	ND
E140A	Glutamine	ND	ND	ND	ND
P214A	Glutamine	30.6 ± 6.1	0.15 ± 0.01	30 ± 2	1.01 ± 0.14
E311A	Glutamine	ND	ND	ND	ND
WT	Hydroxylamine ^b^	2.0 ± 0.4	0.064 ± 0.003	12.8 ± 0.6	0.033 ± 0.005
R4D	Hydroxylamine	ND	ND	ND	ND
Δ13	Hydroxylamine	ND	ND	ND	ND
E140A	Hydroxylamine	ND	ND	ND	ND
P214A	Hydroxylamine	1.5 ± 0.2	0.088 ± 0.003	17.6 ± 0.6	0.059 ± 0.006
E311A	Hydroxylamine	ND	ND	ND	ND

ND, not detectable, ^a^ Parameters determined at a saturating concentration of hydroxylamine, ^b^ Parameters determined at a saturating concentration of glutamine, ^c^ The amount of enzyme used was 5 µM.

## Supplementary Materials

The following are available online at https://www.mdpi.com/2218-273X/10/12/1671/s1. Table S1: Primers used in this study.
