# Supplementary material for: Structural Insight into the Contributions of the N-Terminus and Key Active-Site Residues to the Catalytic Efficiency of Glutamine Synthetase 2
Source: Biomolecules. 2020 Dec 14;10(12):1671. doi: 10.3390/biom10121671 (PMC7764910; doi:10.3390/biom10121671)
Supplement: Supplementary file 1 [file biomolecules-10-01671-s001.pdf]

Supplementary Material

Supplementary Table 1. Primers used in this study

GS gene	Forward primer (5' → 3')	Reverse primer (5' → 3')
WT	CATATGTCCGCTAGGATC	CTCGAGCTATTCGTCCAG
E140A	TGGTTCGGTATTGCGCAGGAGTACACC	GGTGTACTCCTGCGCAATACCGAACCA
P214A	TTCCAGGTGGGAGCCTGCGAGGGCATC	GATGCCCTCGCAGGCTCCCACCTGGAA
E311A	ACCGGAAAGCACGCGACGAGCTCCATC	GATGGAGCTCGTCGCGTGCTTTCCGGT
